# Prevalence, habits and outcomes of using contact lenses among medical students

**DOI:** 10.12669/pjms.346.16260

**Published:** 2018

**Authors:** Nahla Khamis Ibrahim, Hadeel Seraj, Raida Khan, Marwa Baabdullah, Lina Reda

**Affiliations:** 1 *Prof. Nahla Khamis Ibrahim, a- Community Medicine Department. King Abdulaziz University, Jeddah, Saudi Arabia. b- Epidemiology Department, HIPH, Alexandria University, Alexandria, Egypt*; 2 *Hadeel Seraj, Intern, Faculty of Medicine, King Abdulaziz University, Jeddah, Saudi Arabia*; 3 *Raida Khan, Intern, Faculty of Medicine, King Abdulaziz University, Jeddah, Saudi Arabia*; 4 *Marwa Baabullah, Intern, Faculty of Medicine, King Abdulaziz University, Jeddah, Saudi Arabia*; 5 *Lina Reda, Intern, Faculty of Medicine, King Abdulaziz University, Jeddah, Saudi Arabia*

**Keywords:** Contact lenses, Prevalence, Hygienic practices, Complications, Eye infections

## Abstract

**Objectives:**

To determine the prevalence, reasons of use, reported hygienic practices, and complications related to CLs’ usage, and to assess awareness of medical students about CLs’ hygienic behaviours, King Abdulaziz University (KAU), Jeddah, Saudi Arabia.

**Methods:**

A cross-sectional design was done. A multi-stage stratified random sampling was utilized to select 536 medical students. A validated, self-administered, anonymous questionnaire was used. Both descriptive and inferential statistics were applied.

**Results:**

The prevalence of current users of CLs was 40.5%. Females reported significantly much higher prevalence of usage compared to males (OR=8.38; 95% CI:5.2-13.3). Second-year students, and those living in university dormitory had the highest prevalence of CLs usage compared to others. The commonest reason for wearing CLs was cosmetic purpose. Improper CLs-hygienic practices were reported; as exceeding the period required for renewal (45.6%), sharing lenses (16.6%), and sleeping (29.9%), swimming (24.6%) or showering (29.0%) with CLs. Only 16.6% of the participants cleaned their lenses daily. At least one CLs’ related complications was reported by 30.4% of the users. Acute red eye (19.8%), conjunctivitis (18.9%), and corneal abrasion (8.3%) were the commonest CLs’ complications.

**Conclusion:**

A relatively high prevalence of CLs’ usage was found. Participants were aware about CLs-hygienic practices. However, unhygienic CLs-related practices and complications were also reported. Provision of educational messages and training on sound CLs’ hygienic practices are needed.

## INTRODUCTION

The global burden of eye refractive disorders is increasing.^1^ Contact lenses (CLs) have been prescribed since more than a century for correction of refractive errors, cosmetic purpose, and as a therapeutic modality for corneal pathologies.^2^ The use of CLs has greatly increased, and a more increase is expected.^3^

CLs provide safe and effective vision correction^1^ if adequate lenses’ care is ensured as recommended.^4^ However, CLs’ wearers may have risk of eye infections if they fail to wear, clean, disinfect, and store their CLs as directed.^5^ It was reported in 2016 that about 41 million citizens of the USA wear CLs, and more than 99% of them reported at least one behaviour that placed them at risk of eye infection.^4^

A study done in 2013, among the fitters of CLs from 40 counties (2007 to 2011) illustrated an overall increase in the usage of disposable lenses.^6^ The increasing number of CLs’ users resulted in rising of problems attributed to wearing.^7^ A study from the USA revealed that symptoms as burning, itching or tearing eyes were reported among those who continued wearing CLs, and that dry eyes were frequently reported between teen-agers.^8^ A study done in Riyadh, to evaluate prevalence and awareness of female university students and those who attended beauty stores about CLs’ usage revealed that 38.7% of the wearers used CLs without consulting an eye-care practitioner.^9^

Epidemiological studies are needed to provide information on the rate of CLs’ usage, their related behavioural risk factors and complications.^7^ However, little is known about such problem between medical students from Jeddah. So, such study is needed.

The study was performed to determine prevalence, reasons, hygienic practices, and complications related to usage of CLs, and to assess awareness of medical students about CLs’ hygienic practices at King Abdulaziz University (KAU), Jeddah, Saudi Arabia.

## METHODS

A cross-sectional study was conducted during 2016/2017. A multi-stage stratified random sample method was used. Stratification considered genders and educational year (2^nd^ to 6^th^). The sample was calculated by the formula of calculation of sample from cross-sectional study:^10^ The minimal calculated sample was 514, where z = 1.96, p was set at 0.38 9, q=1-p= 0.62, and “d” was set as 0.042.

Participants completed a validated, anonymous, self-administered questionnaire. Both content and face validity were assessed by two experts. Internal consistency reliability was 0.81 using alpha Cronbach’s. The questionnaire asked about personal and socio-demographic information. Awareness of all participants about CLs hygienic behaviours was assessed through 12 questions. History of wearing CLs was determined. The reason of wearing, type & source of lenses were assessed. Reported CLs- behaviors were also looked into. They were also inquired about history of complications.

Statistical analysis was done using Statistical Package of Social Sciences (SPSS) version 21. Descriptive statistics was used. Chi-squared test, Odds Ratios (ORs) and 95% Confidence Intervals (CIs) were calculated. All *p*-values < 0.05 were considered significant.

### Ethical Statement

The study followed ethical standards of Helsinki Declaration. The proposal was approved by the Unit of Biomedical Ethics of KAU, with a “Reference Number of 402-15”. Administrative approvals were taken. A written consent was taken from each student.

## RESULTS

A total of 536 medical students were enrolled in the study, with a slight increase in the sample size for the stratification purpose. About half (50.2%) of the participants have ever tried CLs. The prevalence of current CLs’ users was 40.5%. Most of the wearers (80.2%) used soft lenses. The yearly type of lenses, followed by each of the daily and monthly types were the commonest used lenses.

It was found that 58.9%, 35.9% and 35.9% of the participants used CLs for cosmetics, vision corrections & both purposes, respectively. Optician was the commonest source (92.2%) of CLs. While, 5.1% of the users purchased lenses through internet (5.1%) or from shopping centers (2.8%).

Females used CLs (56.2%) much more than males (13.3%), with highly statistical significant difference (*OR=8.38; 95% CI: 5.2-13.3*). Table-I. The second-year medical students and those lived in university dormitory reported the highest prevalence of CLs wearing (*p* < 0.05).

**Table-I:** Relationship between the current using of contact lenses and the study variables among medical students at King Abdulaziz University.

Contact lenses	Users (217)		None-users (319)		X^2^(p)	OR (95 % CI)

Variables	No	%	No	%		
Gender						
Female	191	56.2	149	43.8	95.01(0.000)	8.38(5.2-13.3)
Male	26	13.3	170	86.7		
***Marital status***						
Single	197	39.7	299	60.3	1.62(0.203)	0.66 (0.3-1.3)
Married	20	50.0	20	50.0		
***Educational year***						
Second	65	56.4	51	43.6	42.12(0.000)	3.36 (1.87-6.06)
Third	29	25.7	84	74.3		0.91 (0.49-1.70)
Fourth	70	54.3	59	45.7		3.13 (1.76-5.57)
Fifth	27	31.4	59	68.8		1.21 (0.63-2.31)
Sixth	25	27.5	66	72.5		1
***Residence***						
With family	201	40.9	290	59.1	6.36(0.036)	2.54 (1.01-6.38)
University dormitory	10	58.8	7	41.2		5.24 (1.39-19.64)
Private dormitory ^(RC)^	6	21.4	22	78.6		1
***Father education***						
University and above	148	39.4	228	60.6	0.66(0.417)	0.86 (0.6-1.2)
Less than university	69	43.1	91	56.9		
***Mother education***						
University and above	136	42.0	188	58.0	0.76(0.385)	1.17(0.8-1.7)
Less than university	81	38.2	131	61.8		
***Father occupation***						
Professional	172	41.1	246	58.9	0.347(0.556)	1.13(0.7-1.7)
Non-professional	45	38.1	73	61.9		
***Mother occupation***						
Professional	100	38.3	161	61.7	0.995(0.319)	0.84 (0.6-1.2)
Non-professional	117	42.5	158	57.5		
***Family income***						
More than Enough	73	37.1	124	62.9	1.66(0.436)	0.69 (0.22-2.12)
Less than enough ^(RC)^	138	42.5	187	57.5		0.86 (0.28-2.62)
	6	46.2	7	53.8		1

RC: Referent Category.

About 7.8% of the users had never renewed their lenses, and 28.6% used the daily disposable lenses. Table-II. Furthermore, 45.6% of the users reported exceeding the period required for CLs’ renewal. Regarding cleaning, 31.8% of the users reported never cleaning their lenses. Regarding lens’ case, 12.9% of the participants reported never cleaning it. Results revealed that 29.5% of the participants reported sleeping with lenses (18.9% for a nap, and 10.6% overnight). It was found that 16.6% of the users shared CLs with others. Water exposure of CLs was reported during showering (29.0%) and swimming (24.6%).

**Table-II:** Awareness of all medical students about contact lenses hygienic behaviours, and their related complications.

Contact lenses’ related complications	Correct answer		Incorrect answer	

	No.	%	No.	%
Increased by sharing lenses	496	92.5	40	7.5
Increased by sleeping with lenses	480	89.6	56	10.4
Increased by non-washing hands prior handling lenses	445	83.0	91	17.0
Increased by non-using fresh cleaning solution	409	76.3	127	23.71
Increased when swimming with CLs	374	69.8	162	30.2
Increased when replacing lenses less frequently than recommended	269	50.2	267	49.8
Decreased when replacing lenses’ cases	240	44.8	296	55.2
Increased when rinsing lenses with tap water	239	44.6	297	55.4
Increased by showering with lenses	239	44.6	297	55.4
Complications are common if hygienic practice are not taken	146	27.2	390	72.8
Increased when adding fresh cleaning solution to existing solution in lens case	122	22.8	414	77.2
Corneal infection can occur as a complication of CLs’ unhygienic practices	349	65.1	187	34.9

About 30.4% of the users reported at least one CLs’ related complication. Table-III. Acute red eye (19.8%), conjunctivitis (18.9%), and corneal abrasion (8.3%), dry eye (4.6%) and abscess (3.2%) were the commonest complications. Each of keratitis and corneal ulcer was reported by 2.8% of wearers. Stye (0.9%) was the least frequent complication.

**Table-III:** Contact lenses-hygienic practices reported by medical students’ users at King Abdulaziz University.

Hygienic practices	No.	Percent
***Renewing contact lenses***		
Daily	62	28.6
Weekly	10	4.6
Monthly	62	28.6
Annually	66	30.4
Never	17	7.8
***Exceeding the recommended period of renewal***		
Yes	99	45.6
No	118	54.4
***Cleaning lenses***		
Daily	36	16.6
Weekly	45	20.7
Monthly	54	24.9
Annually	13	6.0
Never	69	31.8
***Washing hands before putting on lenses***		
Yes	156	71.9
No	61	28.1
***Using soap when washing hands before using lenses***		
Yes	124	57.1
No	93	42.9
***Drying hands before wearing contact lenses***		
Yes	132	60.8
No	85	39.2
***Using makeup with contact lenses***		
Yes	164	75.6
No	53	24.4
***Cleaning contact lens cases***		
Never	28	12.9
With water	60	27.7
With soap	13	6.0
With contact lenses solution	61	28.1
Wear daily disposable lenses	55	25.3
***Replacing the cleaning solution***		
Never	30	13.8
Every night	20	9.3
Most nights	109	50.2
Wear disposable lenses	58	26.7
***Sleeping in lenses***		
Never	153	70.5
Only on a nap	41	18.9
Overnight less than once / month	12	5.5
Overnight ≥ once / month	11	5.1
***Sharing lenses***		
Never	181	83.4
1-2 times in the past	22	10.1
Occasionally	11	5.1
All the time	3	1.4
***Showering with lenses***		
Never	154	71.0
Occasionally	35	16.1
Frequently	9	4.1
All the time	19	8.8
***Swimming with lenses***		
Never	164	75.6
Occasionally	23	10.6
Always, but throw them after	12	5.5
Always and don’t throw them out	18	8.3

Among 217 students used contact lenses

High percentage of the participants correctly identified the increased risk of CLs-related complications in case of sharing lenses (92.5%), sleeping with lenses (89.6%), non-washing hands before handling (83.0%), and non-using fresh cleaning solution (76.0%). Table-IV.

**Table-IV:** Reported contact lenses-related complication(s) by medical students’ users at King Abdulaziz University.

Type of complication	Number (No= 217)	Percent
Any CLs’ related complication (≥ 1)	66	30.4
Acute red eye	43	19.8
Conjunctivitis	41	18.9
Corneal abrasion	18	8.3
Dry eyes	10	4.6
Abscess	7	3.2
Keratitis	6	2.8
Corneal ulcer	6	2.8
Stye	2	0.9
Other complications	9	4.1

Each question was separately asked.

## DISCUSSION

Our results revealed that 50.2% of the participants had tried CLs, which is lower than rate (62%) reported by students from Ohio University, USA.^11^ This difference may be because the previous study was done among all university students.

We found that about two-fifths of our participants were current users of CLs, which coincides with another Saudi study.^12^ However, a lower rate (27.4%) was reported among medical students from Brazil, 2006^13^, which may be due to differences between times of studies, or target populations. Another study among USA adults reported a lower prevalence (16.7%),^5^ than ours, which may be due to differences between target populations.

A predominance of female CLS’ users was illustrated in our study, and this may be due to more usage for cosmetic purposes. This result coincides with other studies.^3^,^11^ The Riyadh’s study reported a higher prevalence of usage (70.2%),^9^ as their study included females only, or due to differences between target populations.

Our study found that 58.9% of the participants used CLs for cosmetic purpose only, which coincides with the results from Riyadh,^9^ and India.^3^ Furthermore, 35.9% of our participants used CLs for visual correction, which is higher than the percentage from Riyadh (19.1%).^9^ Furthermore, 28.6% of our participants used CLs for both cosmetic and visual corrective purposes, which is lower than the rate reported among Pakistani health care providers.^14^ This discrepancy may be due to the differences between ages, and the populations.

Yearly lenses were the commonest used type in our study, which is contrary to other studies (daily or monthly types).^3^,^13^,^15^ This difference may be attributed to lack of times of medical students to frequently purchase lenses. We also found that the majority of our CLs’ users obtained their lenses from opticians. On the contrary most of participants from Sydney purchased them from optometrists.^16^ This difference may be due to availability of optometrists in Australia, or because their study was done through focus group (20 wearers).

The present study revealed that 71.9% of CLs’ users washed their hands before wearing lenses. A higher percentage (89.4%) was reported from Riyadh as they received instructions about CLs’ hygienic practices.^9^ Our study showed that 57.1% of the participants washed their hands with soap and water before touching lenses, which is better than results from Maldives (44.2%).^17^ This discrepancy may be due to differences between target populations.

In Thailand, five improper CLs-care practices were reported as wearing lenses longer than recommended, not changing storage solution daily, swimming with lenses, using tap water for rising lenses and not washing hands before handling lenses.^18^ These results are in line with our findings. Furthermore, our results showed that about half of CLs’ users exceeded the recommended period of renewal, which coincides also with the Maldives’ study.^17^

Overnight lens usage was reported to be associated with much increased risk of microbial keratitis.^19^ In the current study, 29.5% of our participants slept with their lenses (18.9% for a nap and 10.6% overnight). These results are in line with results from Riyadh,^9^ Maldives^17^ and Australia.^20^ However, 50.2% of adults’ wearers from USA online study reported sleeping overnight with CLs.^5^ This discrepancy may be related to differences between target populations or types of lenses. Our study revealed that 18.9% of the students napped with their CLs, which is lower than the rate reported between the adults from USA (87.1% have ever napped with lenses).^5^ This difference may be because many soft and some rigid CLs from the USA have approved indications for sleeping.

Our results showed that 29.0 % and 24.4% of the users reported taking showers and swimming with CLs, respectively, which coincide with results from Maldives.^17^ However, the USA online study illustrated much higher rates (84.9% and 61.0%, respectively).^5^ This discrepancy may be also related to differences between populations.

The percentage of our participants who replaced CLs’ cleaning solution overnight was 9.2%, and those who occasionally replaced it was 50.2%. The study of Riyadh found that percentage of replacement every day to two days was 72.7%.^9^

Proper CLs’ hygienic practices are associated with lower complications.^20^ About one-third of our participants reported at least one CLs-related complication(s). However, a higher rate was reported among university students from Makkah.^12^ This discrepancy may be due to differences between the target populations.

In the current study, acute red eye and conjunctivitis were the comments reported CLs’ complications, while keratitis was reported by only 2.8%. Similar findings were reported from Brazil^13^ and India.^3^ The online study done among USA adults showed that approximately one-third of the users reported occurrence of CLs-related red or painful eye that required visiting physician.^5^ Jones, et al.^8^ reported a slightly higher rate of red eyes between participants aged < 16 years. Such discrepancies may be due to differences between ages. Cho, et al^21^ reported high tear cytokine concentration and conjunctival cell metaplasia in habitual reusable soft CLs’ wearer.

Dry eyes occurred among about 4.6% of our participants, which agrees with the results of Jones, et al.^8^ However, higher rates were reported from the study done in Japan and Canada^22^, and among senior Chinese high school students.^23^ This discrepancy may be also attributed to differences between age groups.

The rate of severe eye complications in our study was much lower than that reported from London hospital’s emergency department.^24^ The cause of such discrepancy may be the differences between both study settings.

Regarding awareness of all participants about CLs-related complications, many of them had good knowledge. On the other hand, results of a recent study from Ghana, 2017, found that only 34.8% of the glass wearers knew about CLs.^1^ The differences between the target populations from both countries may attributed to such differences.^1^

## CONCLUSION

A relatively high prevalence of CLs usage prevailed among medical students in the present study. Females, second-year students, and those who lived in university dorms reported higher prevalence of use. The commonest cause of usage was for cosmetic purpose. Non-compliance with CLs’ hygienic practices prevailed among users. Acute red eye and conjunctivitis were the commonest complications. However, keratitis, corneal ulcer and stye were less frequently occurred. Provision of sound educational messages for all CLs’ consumers by ophthalmologist, optometry and at dispensing shops is needed. Theses educational messages need to concentrate on hygienic practices and complications related to usages.
